# Fragger: a protein fragment picker for structural queries

**DOI:** 10.12688/f1000research.12486.2

**Published:** 2018-04-10

**Authors:** Francois Berenger, David Simoncini, Arnout Voet, Rojan Shrestha, Kam Y.J. Zhang

**Affiliations:** 1 System Cohort Division, Medical Institute of Bioregulation, Kyushu University, Fukuoka, Japan; 2 LISBP, Toulouse, France; 3 Laboratory of Biomolecular Modelling and Design, KU Leuven, Heverlee, Belgium; 4 Department of Systems and Computational Biology, Albert Einstein College of Medicine, Bronx, NY, USA; 5 Structural Bioinformatics Team, Division of Structural and Synthetic Biology, Center for Life Science Technologies, RIKEN, Yokohama, Kanagawa, Japan

**Keywords:** protein fragments, protein design, fragments database, structural query, triangular inequality

## Abstract

Protein modeling and design activities often require querying the Protein Data Bank (PDB) with a structural fragment, possibly containing gaps. For some applications, it is preferable to work on a specific subset of the PDB or with unpublished structures. These requirements, along with specific user needs, motivated the creation of a new software to manage and

query 3D protein fragments. Fragger is a protein fragment picker that allows protein fragment databases to be created and queried. All fragment lengths are supported and any set of PDB files can be used to create a database. Fragger can efficiently search a fragment database with a query fragment and a distance threshold. Matching fragments are ranked by distance to the query. The query fragment can have structural gaps and the allowed amino acid sequences matching a query can be constrained via a regular expression of one-letter amino acid codes. Fragger also incorporates a tool to compute the backbone RMSD of one versus many fragments in high throughput. Fragger should be useful for protein design, loop grafting and related structural

bioinformatics tasks.

## Introduction

Nowadays, a large number of protein structures are available (122,761 as of July 2017 at RCSB) and protein fragments are frequently used in structural bioinformatics. Protein structure prediction methods such as Rosetta ^1^, QUARK ^2^ and EdaFold ^3, 4^ use protein fragments as building blocks. Protein fragments are also used in crystallographic phasing ^5– 7^ and model rebuilding ^8^. The quality of protein models can be improved by combining protein fragments with molecular dynamics ^9^. Other applications include the curation of unresolved loops in crystal structures ^10, 11^, grafting of loop sequences on protein scaffolds and other protein design algorithms ^12, 13^.

When there are too many fragments to search from, an efficient strategy is necessary to reach sub-linear search times. This problem is well-known to the chemoinformatics community, which has developed several efficient strategies to screen large databases of small molecules. For example, geometric embedding and locality sensitive hashing ^14^, kd-trees ^15^, a tree data structure (called *µ*-tree) with a heuristic ^16^, bounds of similarity scores for chemical fingerprints ^17^ and a proximity filter based on the logical exclusive or operator ^18^ have all been developed to this end.

Currently, several fragment pickers ^19– 22^ and protein fragment databases ^23– 28^ are available. Of particular interest is the Super method ^20^ that uses the lower bound of RMSD ^29^ to screen the whole fragment space. However, our research on protein design and refinement of protein decoys for crystallographic phasing required specific options and therefore a new fragment picker.

## Methods

### Implementation

**Algorithm 1. Query with a fragment and an RMSD threshold. Comments are enclosed between braces**

**Input:** *D*: fragment set to query

**Input:** *R*: reference fragment set

**Input:** *q*: query fragment

**Input:** *d _q_*: RMSD threshold

**Output:** *M*: matching fragment set

   
*M* ← *D*

   {fuzzy query: prune the fragment space}

   
**for** *r _j_* in *R* **do**

      
*d* ← *distance*( *q*, *r _j_*)

      
*d _inf_*  ← *d* – *d _q_*

      
*d _sup_* ← *d* + *d _q_*

      { *distance*( *f _i_*, *r _j_*) comes from the database index}

      
*M*  ← {∀ *f _i_* ∈ *M*  | *distance*( *f _i_*, *r _j_*) ∈ [ *d _inf_*, *d _sup_*]}

   
**end for**

   {exact query: refine the result of pruning}

   
*M*  ← {∀ *f _i_* ∈ *M*  | *distance*( *f _i_*, *q*) ≤ *d _q_*}

   
**return** *M*

**Figure 1.**
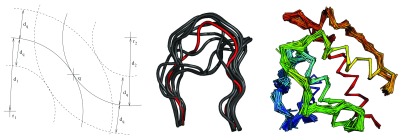
Left: pruning the fragment space for query distance *d _q_* and query fragment *q*. *q* is at distance *d*_1_ (resp. *d*_2_) from reference fragment *r*_1_ (resp. *r*_2_). Only fragments which are both within *d*_1_ ± *d _q_* of *r*_1_ and *d*_2_ ± *d _q_* of *r*_2_ will undergo an RMSD calculation. Middle: 13 residues loops that can connect residue ALA 98 to GLY 110 in chain A of PDB 1MEL. The query loop is shown in red. Only its first and last three residues were used to rank the retrieved fragments. Right: Backbone of PDB 1BKR covered with ten residue fragments from non-homologous proteins retrieved with Fragger.

Fragger exploits the triangular inequality of RMSD ^30^ to prune the fragment space ( Figure 1 and Algorithm 1). RMSDs are computed efficiently via the QCP method ^31^. Fragger is written in OCaml ^32^, except backbone RMSD computations which are performed with a new version of the C++ ranker tool from Durandal ^33^. Computations are parallelized on multi-core computers via the Parmap library ^34^.

Fragger allows a database to be queried with a fragment and an RMSD threshold. Matching fragments are ranked by RMSD to the query. Fragger’s ranker tool allows to compute the backbone RMSD of a single fragment versus many. Fragger can deal with residue gaps or a selection of residues from the query, create a fragment database from a set of Protein Data Bank (PDB) files, work with all fragment lengths and extract specific or randomly-chosen fragments from a database.

Compared to existing fragment pickers, some of the specific functionalities required by users include:

- Outputing only the N best or N first found fragments matching a query (this can make a query terminate faster)
- Constraining the amino acid sequences allowed to match a query (for loop grafting; such filtering is applied after RMSD pruning of the fragment space)
- Reading and writing PDB fragments from/to a binary format (faster than reading/writing regular PDB files)
- Preventing a list of PDB codes from matching a query
- Automatically varying the RMSD threshold to the query until a given number of fragments is reached.

### Operation

Users need to install OPAM and the pdbset command from CCP4 in order to use Fragger.

Details on how to install Fragger and usage examples are provided in the README file of the released software.

## Results and discussion

Tests were performed on one core of a 2.4GHz Intel Xeon workstation with 12GB of RAM running Ubuntu Linux 12.04. The PDB dataset is composed of all proteins determined by X-ray, without highly similar sequences (30% sequence identity cutoff) in order to create a challenging set of fragments to benchmark a protein design algorithm. It contains 13,554 PDBs. PDBs were extracted from the protein databank website using the advanced search tab and ticking the "Retrieve only representatives at 30% sequence identity" box. Querying with a three (resp. nine) residues fragment takes at least 6.75s (resp. 5.2s).

Query times vary with the query fragment, reference fragments, indexed proteins and RMSD tolerance to the query. In general, the longer the required fragment length and the smaller the RMSD tolerance, the faster the query.

Reference fragments can be chosen randomly. Pruning of the search space is better if there are at least three reference fragments, far from each other. Once a RMSD index has been computed for a randomly chosen fragment ( *f _i_*), taking the furthest fragment from it ( *f _j_*) and the median fragment ( *f _k_*) would give three acceptable reference fragments. For interested contributors, some good heuristics can be found in the literature but were not implemented in Fragger, like Brin’s greedy algorithm ^35^.

For one time tasks, it is not necessary to create RMSD indices and actually query a database, as fragments extraction and RMSD computations are fast enough. For example, it takes only 15s to generate all (41,200) fragments of 13 residues starting with alanine and ending with glycine (middle of Figure 1). Ranking them to the query takes 1.5s. When working on PDB files, the ranker tool included with Fragger can compute 66,580 (resp. 23,784) RMSD */*s on the backbone of three (resp. nine) residue fragments. These numbers become 304,149 (resp. 138,744) RMSD */*s when working on Fragger’s binary-encoded PDBs. In the future, it might be possible to improve the performance of Fragger by incorporating a faster score than RMSD, such as BCscore ^36^.

Fragger can be useful for protein design, loop grafting and retrieval of candidates to rebuild low-confidence regions of protein models ^6^.

## Data availability

All data underlying the results are available as part of the article and no additional source data are required.

## Software availability

Fragger can be downloaded from: https://github.com/UnixJunkie/fragger

Archived source code at the time of publication: https://zenodo.org/record/877320

Software license: LGPL.
