# Supplementary material for: Fragger: a protein fragment picker for structural queries
Source: F1000Res. 2018 Apr 10;6:1722. Originally published 2017 Sep 22. [Version 2] doi: 10.12688/f1000research.12486.2 (PMC5773926; doi:10.12688/f1000research.12486.2)
Supplement: Peer review history

# Peer review: Referee response for version 1

**DOI:** 10.5256/f1000research.13520.r28334

**Published:** 2018-01-18

**Authors:** Charlotte Deane, Saulo de Oliveira

**Affiliations:** 1 Department of Statistics, University of Oxford, Oxford, UK; 2 Department of Statistics, Oxford University, Oxford, UK

In this paper, the authors describe Fragger, a web server for retrieving fragments from a  database of structures based on a query fragment. Their program allows for customisation in terms of number of fragments output and in terms of sequence constraints.

 The paper is made up of previously published methods put together as a potentially useful package.

 The Super method (mentioned in the introduction of the manuscript) seems to fulfil the same purpose as Fragger. No comparison is provided between the two methods in terms of performance and quality of fragments output.

 It is unclear from the Methods section how the reference fragment set is selected. It is mentioned in the Results that these fragments can be selected at random, but the authors never discuss whether this choice can have an impact on the performance of the algorithm.

We have read this submission. We believe that we have an appropriate level of expertise to confirm that it is of an acceptable scientific standard.

# Response

**Published:** 2018-04-04

**Authors:** Kam Zhang

**Affiliations:** RIKEN, Japan

> The Super method (mentioned in the introduction of the manuscript) seems

 > to fulfil the same purpose as Fragger. No comparison is provided between

 > the two methods in terms of performance and quality of fragments output.

 Super uses only CA to calculate RMSD.

 Fragger uses backbone atoms to calculate RMSD, since our users want

 to preserve secondary structure information.

 Since Super uses four times fewer atoms than Fragger for each RMSD calculation,we don't think such a comparison would be fair.

 > It is unclear from the Methods section how the reference fragment set

 > is selected. It is mentioned in the Results that these fragments can be

 > selected at random, but the authors never discuss whether this choice

 > can have an impact on the performance of the algorithm.

 Indeed, good reference fragments can improve the search performance.

 We have added a new paragraph and one reference about choosing

 good reference fragments.

# Peer review: Referee response for version 1

**DOI:** 10.5256/f1000research.13520.r26264

**Published:** 2017-10-11

**Authors:** Pierre Tuffery

**Affiliations:** 1 Molécules Thérapeutiques In Silico (MTi) (UMR-S 973), French National Institute of Health and Medical Research (INSERM), Sorbonne Paris Cité, Paris Diderot University, Paris, France

This paper describes an approach to quickly scan large collection of proteins to identify fragments similar to a request. Not considering indels, this approach is, as stated by the authors, in the context of fragment grafting, loop modeling, protein design or crystallographic phasing.

 The metrics used to quantify the similarity is that of the RMSd.

 The rationale here is to to use the triangular inequality of RMSd to setup a two step procedure:

 - decompose the complete set of fragments present in the collection of proteins by as a limited subset of representative fragments

 - quickly identify the representative fragments similar to the query in a way to perform effective pruning of the complete collection of fragments, ensuring not discarding the matching fragments, and then perform a systematic search for the fragments of the classes associated with the matching representative fragments.

 This kind of approach has been used in several contexts and is interesting. The manuscript however could easily be improved.

 Here are some specific comments:

 - The introduction could benefit from a better description of the rationale underlying Fragger, including its use in different contexts. For instance, such a strategy has also been used for the fast similarity search of small compounds.

 - The introduction could benefit from a larger overview of the approaches that have been setup to address questions similar to that of Fragger. There are also a series of web servers focusing on this goal that are not cited.

 - The way the algorithm is described makes it rather uneasy to understand. There could first be  some awkwardness in the notations. For instance, in the algorithm description, blanks between d and b and between r and f could be discarded. Secondly, it could be difficult for a reader to understand the role of the representative fragments, the way they are identified and used from the present description of the algorithm. Probably an additional flowchart or figure to explain it would be welcome.

 - The critical parameters of the procedure are not really identified. What are the effective cutoff values, how do they impact on the search ?

 - It seems Fragger offers possibilities to constrain amino acidd sequences. Is it a prior or a posterior filtering ?

I have read this submission. I believe that I have an appropriate level of expertise to confirm that it is of an acceptable scientific standard.

# Response

**Published:** 2018-04-04

**Authors:** Kam Zhang

**Affiliations:** RIKEN, Japan

> - The introduction could benefit from a better description of the

 > rationale underlying Fragger, including its use in different contexts. For

 > instance, such a strategy has also been used for the fast similarity

 > search of small compounds.

 We have added an extra paragraph in the introduction to mention

 related methods found in chemoinformatics.

 > - The introduction could benefit from a larger overview of the

 > approaches that have been setup to address questions similar to that of

 > Fragger. There are also a series of web servers focusing on this goal

 > that are not cited.

 We have added several citations to web servers and protein fragment databases

 which are using various methods.

 > - The way the algorithm is described makes it rather uneasy to

 > understand. There could first be some awkwardness in the notations. For

 > instance, in the algorithm description, blanks between d and b and

 > between r and f could be discarded. Secondly, it could be difficult

 > for a reader to understand the role of the representative fragments,

 > the way they are identified and used from the present description of

 > the algorithm. Probably an additional flowchart or figure to explain it

 > would be welcome.

 We have renamed some variables in the algorithm to bypass typographic

 problems introduced by the journal's style-sheet.

 We have also added a new paragraph and one reference about choosing

 good reference fragments.

 > - The critical parameters of the procedure are not really identified. What

 > are the effective cutoff values, how do they impact on the search ?

 This is quite complex: the search speed is influenced by the protein

 database, the fragment length and the query RMSD tolerance.

 In the manuscript, we now summarize the general trend as:

 "In general, the longer the required fragment length and the smaller

 the RMSD tolerance, the faster the query.".

 > - It seems Fragger offers possibilities to constrain amino acidd

 > sequences. Is it a prior or a posterior filtering?

 We updated the manuscript to indicate that this is done

 after geometric filtering.
